# Editorial for the COVID-19 special issue of PHCRD

**DOI:** 10.1017/S1463423621000761

**Published:** 2021-12-03

**Authors:** Andrea Canini, Maria van den Muijsenbergh, Diederik Aarendonk, Sally Kendall

The COVID-19 pandemic has confronted us with unprecedented challenges, with devastating loss of life and ill health. Primary healthcare (PHC) professionals worked in the frontline, risking their own health in protecting their patients and communities, especially the most vulnerable, as articulated by Goodyear-Smith (2021) in her editorial for this issue.

Nevertheless, this pandemic has provided us with new opportunities: it can be a game changer to strengthen primary care, as a strong person-centred, community-oriented PHC can be highly effective in promoting and protecting health during a pandemic, as well as preventing and treating disease.

Worldwide, the COVID-19 pandemic has shed a light on the pervasive impacts of social and structural inequities in society, having a disproportionate impact on people who were already disadvantaged because of their ethnicity, age, health status, income, residence or occupation.

In all these groups, a higher infection rate was noticed as well as a higher number of severe cases. The reasons for this are multiple:

Higher workplace and household exposure, higher prevalence of chronic diseases and limited health literacy added to the already existing barriers in accessing healthcare for socially vulnerable groups leading to late presentations and postponed care.

Not only are these groups at higher risk for COVID-19 infection, they also are more prone to suffer from the ‘collateral economic and social damage’ caused by the pandemic. Many low-paid jobs are or will be lost due to the lock-downs, leading to more poverty worldwide; the school closures have increased the educational gaps as many low-income households lack tools for home-teaching, and many low educated parents do not feel up to the task to provide their children with the necessary support for their homework. This all has led to rising physical and psychological distress in these vulnerable populations, as we know from several studies conducted at the global level some of which we publish in this special issue of PHCRD.

What are governing bodies doing with this knowledge, to keep control of the pandemic and at the same time prevent worse health outcomes and postponed healthcare of disadvantaged communities? Whilst the Outbreak Management Teams such as Rajan and colleagues (2020) discuss, politicians, virologists and epidemiologists are dominating, and those who suffer from social and societal consequences are under-represented. The World Health Organisation (WHO, 2020) has drawn global attention to the impact of COVID-19 on food security the complexity of which has a wider impact on employment, livelihoods, food choices and health. These issues are understood by multi-disciplinary PHC teams but not addressed widely by the political debate that focuses on the elimination and prevention of disease rather than the wider determinants.

Hence, our care PHC provision should aim to achieve not only the absence of disease but maximise quality of life for individuals and communities through a Community Orientated (Kassler, 2021; Haldane et al., 2020) approach to primary care. This leads to the need to involve and engage citizens in their care needs and processes, and, furthermore, in designing and co-producing the provision of care.

Adhering to the core values of PHC, we can move towards equity of health and also a rapid and effective response to the pandemic that addresses community health need.

Primary healthcare professionals are used to focusing on person-centred, comprehensive care for their community. Also during this pandemic, they reach out to their vulnerable populations, often in collaboration with their local government and community organisations. For example, they realised care and isolation facilities for the poor or homeless, and they provided understandable information about the infection, preventive measures and vaccination. And their involvement in vaccination programs helped to reach underserved groups, who have little trust in other healthcare professionals or governmental services.

Challenged by the new situation, they rapidly reorganised their practices to ensure continuity of care

Overall, the importance of integration of medical care with public health and social services has again proven to be vital; community engagement is needed to achieve this, (Allen et al., 2020; Mash et al., 2021). Thus, COVID-19 appeared to become a showcase for strong integrated, community-oriented PHC systems

Primary care remains the cornerstone of pandemic response and has shown to be highly adaptable in meeting the unique demands of the pandemic. Primary care needs to be resourced, with adequate equipment, training and financing, as recommended by the 30by2030 campaign and discussed by De Maeseneer (2021) in his editorial for this issue.

We need integration with public health to improve the health of our communities: in addition to person-centredness, we have to acquire a community-oriented perspective. This would be enhanced by the training of primary care social epidemiologists who provide us with the relevant information on health and wellbeing, including the social determinants of health, of our communities and practice populations.

We need an integration of primary care with social care, and to ensure that social causes for health problems can be addressed, we need to stimulate and facilitate inter-professional collaboration

We, doctors, nurses, social workers, pharmacists, physiotherapists, midwives, occupational therapists alike, have to be trained and facilitated in collaborating with each other and with informal carers and patients across communities, empowering and supporting people to promote and protect their health in the face of a wide range of social determinants that underlie the response to a pandemic.

What can we take away from this?

Primary care providers usually know which of their patients are most vulnerable; we must actively get in touch with them.

We need to strengthen our person-centred, community-orientated approach, improving the ability of people to recognise their strengths, seek appropriate help and self-manage their chronic conditions and decrease their dependency on health services, whilst continuing to support people in times of need. This also means that PHC professionals are there to provide compassionate care and support at the end of life too, a consequence of the COVID-19 pandemic that has swept the globe and left millions without their loved family members and friends. Strong community-orientated primary health care is there for our populations during all stages of the life cycle.
